# Papaya (*Carica papaya* L.) Flavour Profiling

**DOI:** 10.3390/genes12091416

**Published:** 2021-09-15

**Authors:** Ziwei Zhou, Rebecca Ford, Ido Bar, Chutchamas Kanchana-udomkan

**Affiliations:** Centre for Planetary Health and Food Security, School of Environment and Science, Griffith University, Nathan, QLD 4111, Australia; ziwei.zhou2@griffithuni.edu.au (Z.Z.); i.bar@griffith.edu.au (I.B.); c.kanchana-udomkan@griffith.edu.au (C.K.)

**Keywords:** papaya breeding, flavour profiling, biosynthesis pathways, gene identification

## Abstract

A major challenge to the papaya industry is inconsistency in fruit quality and, in particular, flavour, which is a complex trait that comprises taste perception in the mouth (sweetness, acidity, or bitterness) and aroma produced by several volatile compounds. Current commercial varieties vary greatly in their taste, likely due to historical prioritised selection for fruit appearance as well as large environmental effects. Therefore, it is important to better understand the genetic and biochemical mechanisms and biosynthesis pathways underpinning preferable flavour in order to select and breed for better tasting new commercial papaya varieties. As an initial step, objectively measurable standards of the compound profiles that provide papaya’s taste and aroma, together with ‘mouth feel’, are required. This review presents an overview of the approaches to characterise the flavour profiles of papaya through sugar component determination, volatile compound detection, sensory panel testing, as well as genomics-based studies to identify the papaya flavour.

## 1. Introduction

Papaya (*Carica papaya* L.) is one of the top five most commonly grown tropical fruit crops throughout tropical and subtropical regions worldwide, including in Australia, Hawaii, and Southeast Asia [1]. Papaya fruit is juicy with a sweet flavour and the ripe fruit is rich in vitamins A and C, folate, as well as calcium [2]. The fruit is valued for its nutritional status and is usually eaten raw, whereas unripe green fruit can be eaten both raw and cooked, for example, in green papaya salad. In addition, unripe papaya is a source of papain, an endolytic plant cysteine protease that plays a crucial role in many vital biological processes in all living organisms and that has been used in meat tenderising for thousands of years [3,4]. 

Due to its high productivity, nutritional value, and functionality, papaya has become an important commercial fruit crop worldwide. The global production of papaya for the past twenty years has steadily increased, mainly because of increased production in India and demand by the United States, reaching a peak in 2016 of 13.09 million tonnes. In 2018, 60.9% of the world’s total papaya production was in three countries: India (138 thousand ha and 5.99 million tonnes), Brazil (27.2 thousand ha and 1.06 million tonnes), and Mexico (18 thousand ha and 1.04 million tonnes) [5]. 

Although great gains have been made in increasing fruit yield, there has generally not been a simultaneous improvement in flavour quality. This has likely led to reduced market uptake, hence recent breeding has focused on improving fruit and flavour quality traits with the intention to expand the market [6]. Fruit quality comprises several important factors including flavour, nutrition, appearance, texture, and postharvest processing. Flavour is a complex trait that includes taste perception in the mouth (sweetness, acidity, and/or bitterness) and aroma, which is produced by several volatile compounds [7]. Therefore, the combination of both mouth perception as well as amounts and ratios of volatile compounds present in the flesh plays a major role in determining the perception and acceptability of papaya flavour by consumers. Together, these considerations are important in strategic breeding, branding, and marketing of premium papaya cultivars to align with consumer acceptance and demand. To achieve this, objective standards of good taste and aroma must be set. Additionally, molecular markers for detecting desirable fruit traits at an early stage of papaya growth are needed for targeted genomics-assisted breeding strategies. The following is a review of the key factors and current knowledge in papaya fruit flavour quality and the considerations for the strategic breeding of the flavour of preference.

## *2.* Papaya Flavour

Many factors, including the biochemical and environmental contributions to flavour profiles, how these are perceived, and the suitable eating stage, must be understood to successfully breed papaya and improve its flavour. Fruit flavour comprises sugars, acids, and volatile components [8]. The combination of a consumer’s mouth perception together with an understanding of the preferred acidity, sweetness, and known amounts and ratios of specific volatile compounds can be used to develop a tool to differentiate and select for flavour types or profiles. In this review section, sugar accumulation and volatile compounds, as well as the genes involved in fruit flavour metabolism pathways in papaya, will be discussed.

### 2.1. Sugar Accumulation and Detection

Sugars contribute largely to fruit taste as sweetness is one of the major factors in determining papaya flavour and hence fruit quality [9]. In order to breed for preferred sweetness types and levels, knowledge on the way sugars are metabolised and stored in fruit post-harvest is required. Accordingly, a model of metabolism and the accumulation of sugars translocated to fruit was summarised by Yamaki [10]. Four physiological steps, unloading, membrane transport, metabolic conversion, and compartmentation, were identified to determine the translocation of sugars from leaves to fruit and referred to as ‘sink strength’. Photoassimilates are firstly synthesised in leaves through photosynthesis and then used to form sugars including sucrose and sorbitol. After this, sugars are transported from the phloem to fruit tissue following the ‘pressure flow’ theory. Sugars are then unloaded into fruit cells through symplasmic and apoplasmic routes and metabolised into diverse substances by several enzymes. Finally, sugars in cells are accumulated and compartmented in vacuoles [10,11,12,13]. Among these four steps, metabolic conversion is the most important stage for sugar accumulation. The key enzymes involved in this step are soluble acid invertase, insoluble acid invertase, neutral invertase, sucrose phosphate synthase (SPS), and sucrose synthase (SS) [10,11,14,15,16]. Among these, acid and neutral invertases catalyse the hydrolysis of sucrose to fructose and glucose; SPS is associated with sucrose synthesis; and SS functions in sucrose synthesis and cleavage [15].

**Figure 1:**
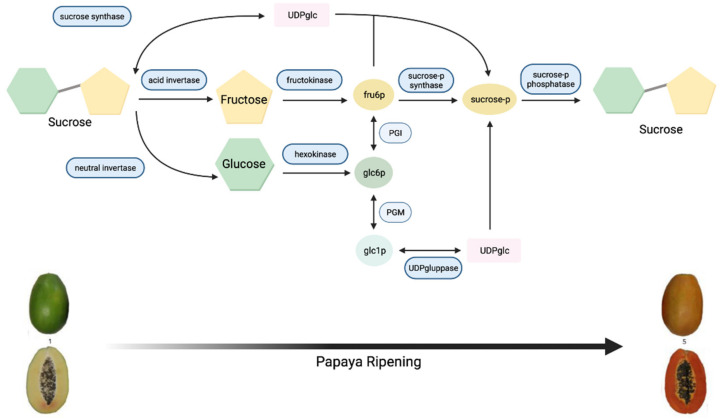
Metabolic pathway of sugar metabolism in papaya fruit, indicating enzyme reactions.

Papaya sweetness is contributed to by three main soluble sugars: glucose, fructose, and sucrose [15,17,18,19]. Accordingly, for papaya sweetness evaluation, it is essential to quantify total sugar content and the ratio of each type of sugar in that total [19,20]. During papaya fruit development, sugar accumulation initiates after seed maturation with the increasing activity of SS, and glucose is the major soluble sugar [15,21]. The metabolic pathway of sugar metabolism and associated enzymes in papaya is shown in Figure 1.

Papaya fruit that is grown for commercial purposes is optimally detached from a tree ~110 days post-anthesis (dpa); however, the flesh is still hard and not acceptable for ripe consumption at this stage [20]. After harvesting, fruit continues to ripen, and a modification in the sugar profile occurs whereby sucrose becomes the predominant sugar, which leads to flesh sweetening [21]. The sucrose pathway is predominantly governed by the activities of SPS and SS, leading not only to sweetness but also changes in cell wall composition and hence a softening in flesh texture [11,15,17,20]. Therefore, SS and acid invertase are the key enzymes in determining papaya sugar accumulation during the early and late fruit development phases, respectively [15]. In 1991, Hubbard’s team [11] conducted a sucrose accumulation analysis of papaya and determined that acid invertase activity is very high during the papaya ripening stage. This finding was achieved by assessing carbohydrate concentrations and activities of SPS, SS, and acid and neutral invertases in fruit at six days after purchase (dap). At this time point, the soluble carbohydrate concentrations and SPS and SS activities did not significantly change. However, the acid invertase activity greatly increased from 60 to 1050 μmoL h^-1^ (g fresh weight)^-1^, indicating a major role in sucrose accumulation after harvest. 

Invertases were also involved in the accumulation of glucose and fructose in ripe papaya fruit variety ‘Golden’ [22]. SPS plays an important role in metabolising neutral sugars, especially mannose from cell walls for the continuous synthesis of sucrose and galactose, the main source of carbon during the synthesis of sucrose [20]. Galactose is metabolised rapidly under high SPS activities, liberating simple sugars, and contributes to sweetness [23]. In addition, several quantitative trait loci (QTLs) for flesh sweetness have been detected in papaya varieties ‘RB2’ and ‘Sunrise Solo’, which are associated with the SS gene family [9,24]. Nantawan et al. [9] determined that sucrose was the main sugar in these two papaya varieties at the ripening stage, contributing 40–60% of the total sugar. 

Fruit sugar content may be detected via high-performance liquid chromatography (HPLC) [25,26], gas chromatography (GC) [27,28], and enzymatic analysis [18,24]. Comparing these techniques, although HPLC and GC are accurate, they are time consuming in their need for specific sample preparation, and the equipment is relatively expensive. On the other hand, enzymatic assays, which measure soluble sugar concentrations based on enzyme metabolism, are far simpler and require just a spectrophotometer. In recent years, biosensor techniques were combined with enzymatic assays to improve the sensitivity and specificity of specific sugar type detection [29]. 

Fruit sweetness is traditionally measured as the unit Degrees Brix (°Bx), which indicates the total soluble solids in solution. One degree Brix represents 1 gram of sucrose in 100 grams of solution, as well as the strength of the solution as a percentage by mass. Brix can be measured according to specific gravity, refractive index, and infrared absorption.

### 2.2. Volatile Flavour-Related Compounds

The identification and quantitation of key volatile flavour metabolites are essential to capture the unique and discriminative characters of pre-identified fruit flavours. These may be generated as chemical fingerprints of the principal sensory flavour identities [30]. The aroma-induced flavour of each fruit may be distinct and dependent on the combination of types and amounts of volatile compounds, as well as the perception thresholds of individual volatile compounds by the taster [31]. In principle, analysis of these compounds could lead to the development of stable biomarkers that could act as chemical fingerprints to accurately and reproducibly describe and subsequently select for flavour preferences. Such biomarkers may also potentially be used to brand and market the key preferred flavour types. 

#### 2.2.1. Volatile Extraction and Detection

The volatile components of papaya have been identified within several studies over the past 50 years using various methods [32,33,34,35,36,37,38]. The first volatile research in papaya was conducted in 1965 by Katague and Kirch [39], who extracted volatiles from two papaya fruit sources, one grown under greenhouse conditions and another obtained from a commercial market. They extracted the volatiles using steam distillation saturated with sodium chloride and an ethyl ether extraction. The compounds were then characterised by thin-layer chromatography and five volatile compounds were detected unique to greenhouse-grown fruit (propyl alcohol, propyl acetate, isopropyl alcohol, amyl alcohol, and methyl amyl ketone), highlighting potential impacts of environmental conditions on volatile production. 

Natural fruit flavours may be divided into two categories: the main odour components (mainly derived from C1, C2, and C5 alcohols and their esters) and the supporting components (derived from C3, C4, and C6 alcohols and their acetates) [7]. Capturing key volatile compounds using a distilling process has limitations of sensitivity, hence sample processing methods were subsequently improved in 1973 by Chan Jr et al. [33], who were able to detect more aroma-related volatiles from pureed flesh than from fresh cut fruits. Of these, chemicals such as linalool, butyric, hexanoic, and octanoic acids and their methyl esters, which were not able to be captured in previous sampling processes, were detected and proposed to be responsible for strong off-odours and off-flavours [38].

Although papaya processing methods were improved, the trace volatile components were still not able to be clearly detected due to the lack of a sensitive detection device. The identification of aroma substances was performed in two steps: (1) Separation of the analytes from the background sample matrix via volatility and/or solubility; and (2) Identification of analytes. Therefore, it is important to choose a method that can extract volatile compounds and eliminate interfering signals from the matrix as much as possible [8]. For this, head space (HS) analysis via solid phase micro extraction (SPME) coupled with gas chromatography–mass spectrometry (GCMS) was developed [40]. SPME is a low cost, sensitive, high speed, solvent-free, and easy to use technique for the extraction of analytes from gaseous, liquid, or solid samples [40]. SPME is a two-step process: (1) Separation of the extraction phase from the sample matrix; and (2) Thermal desorption of the concentrated extracts for transfer directly to an analytical instrument [41]. SPME usually functions as an automatized fibre injection system combined with chromatographic separation modules for the analysis of flavour compounds. This enables the extraction and isolation of all relevant aroma compounds with a good recovery and also allows for the identification of trace analysis components in complex matrices [40,41,42]. Several studies have used SPME coupled with GCMS to analyse volatile compounds in a variety of fruits such as apricot [43], pineapple [44], strawberry [45], apple [46], mango [47], and recently in papaya [38]. 

Almost 400 volatiles were originally identified in papaya [37] using a range of separation techniques such as headspace, gas chromatography, odour olfactometry, and mass spectrometry [8,34,38,48]. Based on these previous studies, the characteristic aroma of papaya fruit is proposed to be produced by combinations of alcohols, esters, aldehydes, and sulphur compounds [49]. 

#### 2.2.2. Odour Thresholds

The perception of fruit flavours by humans is influenced by multiple sensory factors, while aroma compounds are mainly detected by the olfactory system [50]. Humans have approximately 350 olfactory receptor genes for diverse flavour recognition; however, volatile compounds must reach a certain quantity (concentration threshold) to be perceived. Baldwin et al. [51,52] assessed relationships between volatile compound concentrations and sensory descriptions of tomato to determine odour thresholds. Although more than 400 aroma volatiles were reported in tomato, only 15–20 are determined to be present in sufficient concentrations to impact on flavour perception within the human olfactory system [52]. 

Similarly, among 118 aroma compounds identified in the papaya variety ‘Red Maradol’, only 25 were determined to be odour-active and to contribute to the distinctive flavour [45]. Of these, ethyl butanoate, benzyl isothiocyanate, 1-hexen-3-one, (E)-β-ionone, and methyl benzoate were the most odour-active with the highest detection frequencies observed via SPME analysis [37,48]. Similar studies were performed in other fruit including muskmelon [53], mango [54], strawberry [55], and banana [56]. Within these, fruit had a small number of high-impact compounds that dominated the flavour perceptions. For example, in banana (*Musa acuminata* Colla), 3-methylbutyl acetate dominated the ‘ripe’ flavour [56,57]. In other fruit, a complex profile of compounds provided a unique flavour. For example, a combination of cis-3-hexenal, cis-3-hexenol, hexanal, 1-penten-3-one, 3-methylbutanal, trans-2-hexenal, 6-methyl-5-hepten-2-one, methyl salicylate, 2-isobutylthiazole, and β-ionone, each at their individual concentrations, created a ‘fresh ripe’ tomato aroma [52,58].

### 2.3. Consumer Acceptability

To expand and sustain a firm share in today’s competitive fruit marketplace, the major challenge to the papaya industry is to produce attractive fruit aromas and tastes that appeal to most consumers. Although instruments can precisely evaluate biochemical compositions and concentrations, consumer preference, on the other hand, is subjective and may vary within the population. Sensory panel testing and consumer acceptance surveying are required to determine flavour attributes driven by consumers and factors influencing their purchasing decisions. Sensory panel testing also provides imaginable descriptions for flavours. Accordingly, Lieb et al. [38] conducted a sensory panel testing of four papaya varieties (‘Criolla’, ‘Pococí’, ‘SH-5′, and ‘Silvestre’) and the flavour attributes were divided into two groups, orthonasal and retronasal. Both groups included flavour descriptions such as ‘fruity’, ‘tomato-like’, ‘sweaty/rancid’, ‘honey-like’, ‘floral’, ‘cress-like’, ‘pumpkin/melon-like’, and ‘carboard-like’. Trained assessors, who passed a fragrance matching test according to Meilgaard’s sensory evaluation techniques [59], evaluated and scored from the intensities of each flavour descriptor on a 0 to 10 scale. Consequently, correlations between biochemical profiles and human sensory perceptions were made and compared to reference controls. The results pointed out that the significant increasing concentrations of terpenes and lactones are supposed to be involved in the productions of ‘fruity’, ‘flora’, and ‘honey-like’ aromas in the ‘SH-5′ cultivar.

Meanwhile, wider consumer acceptability is relatively more subjective than sensory panel testing and is usually evaluated by large untrained groups via questionnaires. This approach enables the ability to consider potential surveyed population impacts such as cultural origin or preference. Accordingly, a study of consumer preferences of fresh table-ripe papaya was investigated in the Philippines by del Carmen et al. [60]. From their study, a total of 232 consumers, with ages ranging from 40 to 59 years old, were surveyed. The questionnaires were distributed in a supermarket and compromised two parts. The first part evaluated the socio-demographic characteristics of papaya consumers, and the other part determined the consumers’ purchasing attitudes and attribute preferences. Based on their research, the top four papaya attributes determined to influence papaya purchase decisions were sweetness, overall quality (external and internal qualities), colour, and price. Lieb et al. [38] also performed consumer acceptance testing and focused only on the overall acceptance of the papaya samples. Untrained assessors were asked to rate papaya varieties on a scale ranging from “disliked extremely” (0), “neither liked nor disliked” (5) to “liked extremely” (10). Based on this study, the fruit from hermaphrodite plants (‘Criolla’ with a score of 7.1 ± 2.4 points and ‘Pococí’ with a score of 7.0 ± 2.2 points) were significantly more preferred than fruit from female plants (‘SH-5′ with a score of 6.0 ± 3.0; ‘Silvestre’ with a score of 4.3 ± 2.6). Similar to the trained panel results, the consumer preferences may then be correlated with the evaluation of other physicochemical characteristics as well as volatile compound concentrations. A combination of the methods used in these two studies [38,60] may aid in the further analysis of consumer preferences focusing on papaya flavour.

## 3. The Genomics of Fruit Flavour

The genes that contribute to fruit flavour are involved in the production of sugars, acids, and volatile components, which are also involved in other primary and secondary metabolic pathways [6]. Therefore, genomics-based studies to identify the genes controlling flavour and the subsequent development of selective molecular markers have focused on understanding those within the sugar and volatile syntheses pathways [6,9,61].

To select papaya genotypes with high sugar content phenotypes, several of the functional genes related to sugar synthesis and accumulation pathways have been identified [9,24]. As mentioned in the previous section, invertase, SPS, and SS are the key enzymes involved in papaya sugar production throughout the ripening stage [10,11,15,16,17]. Genes involved in the production of these key enzymes have been investigated and identified in a variety of fruits including tomato [62], pineapple [63], apple [64], and papaya [24]. In some fruits, such as tomato [65], citrus [66], and sugarcane [67], SPS activity is directly linked to sucrose accumulation during the fruit maturation stage, but the detailed mechanisms are still unknown. Meanwhile, in other fruits, such as grape berries [68] and pineapple [63], the mechanism for sucrose production is more complicated and regulated by multiple genes.

**Figure 2:**
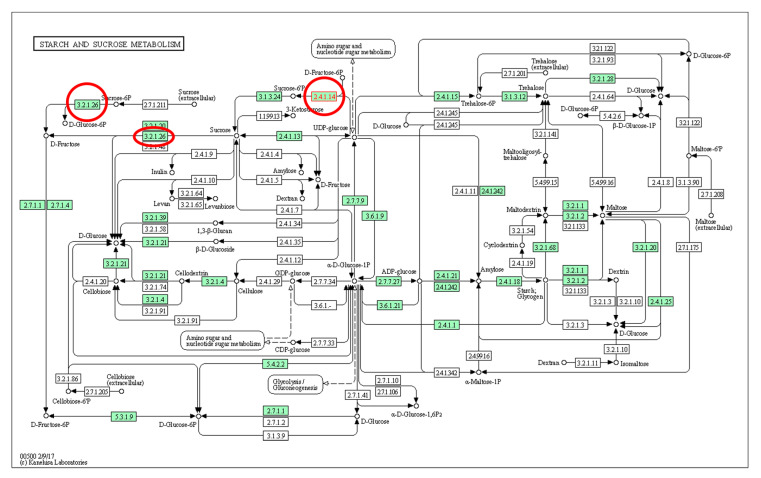
Starch and sucrose metabolism pathway within which the locations of gene *cpSPS2*, *cpCWINV1*, and *cpAVIN2* are labelled in red circles. *cpSPS2* functions at site 2.4.1.14 in *Arabidopsis thaliana*; *cpCWINV1* functions at site 3.2.1.26 in *C. papaya* L.; *cpAVIN2* functions at site 3.2.1.26 in *Solanum* *lycopersicum* [72].

Several genes have been identified for the improvement of fruit sweetness in papaya, including nine predicted to control sugar synthesis and sugar transportation [69]. Among these, two were papaya cell wall invertase genes (*CpCWINV1* and *CpCWINV2*), which have the Glycohydrolase Family 32 (GH32) domain and are responsible for exporting sucrose from the phloem to the cell as well as hydrolysing sucrose into fructose and glucose. A further four were SS genes (*CpSUS1* to *CpSUS4*) and all had sucrose synthase and glycosyl transferase (GT4) domains, which catalyse the reversible conversion of sucrose and UDP to UDP-glucose and fructose. Another three were SPS genes (*CpSPS1*, *CpSPS2*, *CpSPS3*) and all had GT4 and SPS domains. These genes were selected as sweetness candidate genes and identified in two papaya genotypes ‘RB2′ and ‘Sunrise Solo’ by Nantawan et al. [24]. Higher expression levels of *cpSPS1*, *cpSPS2*, *cpSPS3*, *cpSPS4*, *cpCWINV1*, and *cpAVIN2* were observed in ‘Sunrise Solo’, the genotype with a high sugar content (Figure 2). This indicated major putative roles of these genes in sugar synthesis in papaya [24]. A sugar transporter gene (*AT3G05165*) was also discovered in ripe papaya (cv. ‘Golden’) by Fabi et al. in 2012 [70]. Additionally, nine putative enzymes associated with sugars/sugar alcohols with unigenes were identified in ripe papaya (cv. ‘Eksotika’) by using mRNA paired-end sequencing [71]. These were α-galactosidase, α-glucosidase, myo-inositol monophosphatase, β-fructofuranosidase, xylose isomerase, fructose biphosphatase, fructose biphosphate aldose, ribose 5-phosphate isomerase, arabinose kinase, and β-glucosidase. 

These findings contribute to the further identification of possible chemical biomarkers to genetically improve papaya fruit sweetness. In addition, three candidate genes functioning in the regulation of developmental growth (Non-canonical poly(A) RNA polymerase PAPD5 and KIN17-like protein) and protein transmembrane transporter activity (Transmembrane emp24 domain-containing protein P24-beta2) were detected in the QTL maps related to flesh sweetness [9]. The discovery of sweetness-related genes from QTL mapping also plays a major role in identifying functional sequences underpinning the papaya sweetness trait, which, in turn, contributes to the further development of markers for selective breeding strategies.

Volatiles can influence the perception of sweetness and vice versa [73]. Fruit-related volatiles are mainly derived from three different secondary metabolic pathways; (1) the isoprenoid biosynthesis pathway that produces mono- and sesquiterpenes, (2) the shikimic acid aromatic amino acid biosynthesis pathway that produces phenylpropanoids and benzenoid fragrances, and (3) the acyl lipid catabolism pathway that forms short branched-chain aldehydes, alcohols, esters, and ketones. The genes within these pathways represent targets for studies focused on uncovering differential sequence identification, differential expression, and the development of biomarkers for preferred volatile selections [61]. The key flavour volatile genes are generally divided into two classes: (1) those encoding enzymes responsible for the synthesis of the end product and (2) those encoding factors that regulate the synthetic pathways [61]. In papaya, the main contributors to aroma are methyl and ethyl ester derivatives of lipid catabolism [74]. Accordingly, 14 papaya sequences were predicted to be associated with enzymes involved in fruit volatile biosynthesis. Among these, five were highly similar to enzymes in the acyl lipid catabolism pathway, and two were enzymes that encoded the final steps of the fatty acid degradation to acetyl-coenzyme A pathway: Abnormal inflorescence meristem 1/fatty acid multifunctional protein (AIM1) and 3-ketoacyl-CoA thiolase via the β-oxidation pathway [74]. An additional two genes (*CpPALDS2* and *CpPALDR1*) were involved in volatile production from phenylalanine as identified by [69].

Meanwhile, carotenoids play an important role in forming flavour and fragrance volatiles, including aldehydes and ketones in papaya. The four major carotenoid compounds identified in papaya were β-cryptoxanthin, β-carotene, lycopene, and zeaxanthin [75]. The carotenoid profiles were characterised in two papaya cultivars (‘Sui hong’, which is red-fleshed, and ‘Sui huang’, which is yellow-fleshed) using high-performance liquid chromatography ApCI–mass spectrometry (HPLC-ApCI-MS) analysis [75]. High expression levels of *CpCCD1* were noted in ‘Sui hong’, predicted to stimulate the degradation of β-carotene and lycopene and resulting in the formation of a pleasant balsam aromatic odour. Most recently, *CpLIS1* and *CpP450-2* were identified to be responsible for linalool and linalool oxide biosynthesis separately [76]. Meanwhile, *CpAAT1* was shown to catalyse specific esters (trifluoroacetate-trans-2-dodecen-1-ol and trifluoro nonyl acetate), and *CpACX1* was positively associated with the biosynthesis of lactones (δ-caprolactone and δ-decanolactone) during fruit ripening [76]. These chemicals are components of floral and sweet aromas in fruits and vegetables. The mentioned functional gene studies are summarised in Table 1. In subsequent studies, these may be assessed for their correlation with biochemical and sensory evaluations from the same papaya samples.

In addition, the genomic locations of the most active and heritable genes contributing to preferable flavours may potentially be identified through genome mapping approaches. Accordingly, 30 quantitative trait loci (QTLs) governing volatile production in red-ripe tomato (‘*Solanum lycopersicum*’ and ‘*S. Habrochaites*’) were characterised in the tomato genome [77]. The functions of the candidate genes that were co-located within the peaks of the largest QTL were then significantly associated with emissions of apocarotenoid volatiles (geranylacetone and MHO), leucine and isoleucine-derived volatiles, C5 or C6 volatiles, and phenolics [77]. Similarly, sequences within 72 QTLs were associated with the expression of 23 volatile organic compounds in peach [78]. Among these, three QTLs were significantly associated with the production of major aroma compounds: nonanal, linalool, and p-menth-1-en-9-al. Interestingly, both nonanal and linalool were also major volatile compounds detected in many papaya varieties, contributing to citrus-like flavour and floral odour [8,34,38,48]. Comparative analyses with these volatile compounds and their QTLs may provide tools for biosynthesis pathway analysis of these compounds in papaya.

## 4. Gene Identification and Functional Validation

The identification and characterisation of gene sequences related to papaya sweetness and volatile compounds are important for future breeding, branding, and marketing of premium papaya cultivars to align with consumer acceptance and demand. The first step towards this is the identification and functional validation of these genes/sequences within current papaya commercial varieties and advanced breeding lines.

RNA sequencing (RNA-Seq), otherwise known as transcriptomics, is one possible approach to investigate gene/sequence function for identifying those underpinning papaya flavour. This is a sensitive and differential expression approach capable of uncovering important genomic contributors that may be expressed at relatively low levels [61,79,80,81]. There are four stages in the analysis of RNA-Seq data: (1) Alignment and assembly of sequencing reads via an alignment tool such as STAR [82]; (2) Quantification of transcript abundance; (3) Filtering and normalisation; and (4) Differential expression modelling [83].

Meanwhile, quantitative PCR (qPCR) is a reliable and high-throughput method for expression validation and the precise quantification of gene expression. To date, the combination of RNA-Seq and qPCR technology has been applied to study ripening-related genes/sequences, colour-related carotenoid biosynthesis genes, and ring spot virus (PRSV) resistance genes in papaya [84,85,86]. In addition, RNA-Seq has been employed to assess sequences related to fruit volatile compound production and in particular the ‘peach-like’ aroma in strawberry [87,88]. For this, the RNA of ripe and unripe wild strawberry (*Fragaria. Pentaphylla*) was extracted, sequenced, and aligned. The differentially expressed genes (DEGs) present in the ‘peach-like’ flavoured strawberry were then identified and mapped to the KEGG database (www.Genome.jp/keg/, accessed on 29 May 2021) to define contributory genomic mechanisms and pathways. The differential sequences were also correlated with volatile data obtained from GCMS. Based on RNA-Seq analysis, four genes were significantly up-regulated in red fruits and were associated with the lipoxygenase (LOX) pathway, resulting in the deoxygenation and reduction of aldehydes in red fruit. This correlated very well with the GSMS analysis outputs [88]. Aldehydes were termed ‘green-leaf’ volatiles, with decreasing concentrations as the fruit ripens. Since wild strawberry has more diverse aroma patterns than cultivated varieties, the combination of RNA-Seq and GCMS analysis in wild strawberry may provide more information about volatile synthetic pathways, and thus help in the future breeding of cultivars with preferred aromas.

Therefore, RNA-Seq and qPCR analyses together with biochemical analyses may be a suitable combined approach for uncovering the genomic components of papaya flavour. An immediate limitation to RNA-Seq analysis in papaya, however, is the lack of a dense coverage and fully annotated papaya reference genome. This is surprising since the papaya genome size is relatively small (372 Mbp) when compared with other plants such as banana (875 Mbp), plum (883 Mbp), and avocado (883 Mbp) [89]. The first draft genome of papaya was released by Ming et al. (2008), who sequenced the transgenic variety ‘SunUp’, the first commercially available and virus-resistant transgenic papaya, by using Sanger sequencing technology followed by BAC-end sequences as well as physical and genetic maps [79]. To date, this stands as the most comprehensive published papaya reference genome (http://www.plantgdb.org/CpGDB/, accessed on 29 May 2021), yielding 1.6 million high quality reads from a total of 2.8 million whole genome shotgun (WGS) sequencing reads assembled into 271Mb contigs and representing around 75% of the papaya genome. A total of 24,746 genes were accurately annotated from this following the TIGR Eukaryotic Annotation Pipeline [90]. This was a 11–20% lower gene count than detected in Arabidopsis [91] and 34% less than in rice [92]. A denser and better annotated genome assembly is much needed for future advanced papaya genomics studies.

In order to provide further evidence of the functional relatedness of genomic sequences with traits of interest, site-directed gene mutation studies are possible. This includes the use of a clustered regularly interspaced short palindromic repeats (CRISPR)/CRISPR-associated protein9 (Cas9) (CRISPR/Cas9) system. This approach has been applied to modify genomic sequences to achieve desired traits or silence unwanted traits in many crop organisms [93,94]. 

CRISPR/Cas9 vector systems have been widely used in the efficient editing of plant genomes including for gene knockout, gene knock in, and the suppression of virus infection [93]. In rice, Shimatani et al. [95] induced three multiple-herbicide-resistance point mutations (C287T, G590, and W483), which conferred resistance to the herbicide imazamox (IMZ). By mutating these genes in rice, they were able to induce 3.41% IMZ tolerance. In papaya, CRISPR/Cas9-mediated mutations of S-genes have been developed to enable resistance to papaya ringspot virus (PRSV) [96,97]. However, the CRISPR/Cas9 approach requires a robust transformation system and works best when a clear and single target is identified. 

Conversely, flavour is a very complex multigenic trait, comprising numerous reactions mediated by multiple enzymes and genes. Flavour is also likely influenced by several physical and environmental factors, which can lead to qualitative and quantitative changes in biochemical component production [98].

The quantitative variation of floral aroma volatiles in tomato (*Solanum lycopersicum* L.) fruit has been assessed using CRISPR/Cas9 [98]. Accordingly, phenylalanine-derived volatiles (PHEVs) were mapped at major QTLs, leading to the characterisation of FLORAL4 as a candidate gene in PHEV regulation. Subsequently, a CRISPR/Cas9 construct was designed to mutagenize FLORAL4 and the M1 generation was assessed for homozygous mutations and volatile analysis via GCMS. Consequently, decreases in FLORAL4 expression, PHEV reductions, and specific sequence changes were correlated. The same reverse-genetics approaches could be used to identify the functionally relevant sequences for aroma and flavour preference sequences and biochemical compounds in papaya.

## 5. Conclusions

A significant challenge to the global papaya industry is the production of fruit with consumer-driven preferred flavour and aromas. To alleviate this, a targeted breeding approach may be undertaken that initially involves a deep understanding of what constitutes flavour and aroma preference. This is possible through a multi-pronged approach including genomic, biochemical, and sensory profiles and subsequent multi-component correlations. Once genomic sequences have been uncovered that are significantly and robustly associated with key aromas and flavour, these may be assessed for advanced molecular breeding purposes. This would include assessing within multiple environments and across diverse genotypes to ensure any selected genomic components are sufficiently conserved for ongoing selection purposes and sufficiently discriminant to select only the preferred flavour and aromas.

## Figures and Tables

**Table 1:** Summary of key genes forming fragrance volatiles related to carotenoids in papaya.

Genes	Related Volatiles	Papaya Varieties	Reference
*CpCCD1*	β-carotene and lycopene	Sui hong	[75]
*CpLIS1*	Linalool and linalool oxide	Hong fei	[76]
*CpP450-2*	Linalool and linalool oxide	Hong fei	[76]
*CpAAT1*	trifluoroacetate-trans-2-dodecen-1-ol and trifluoro nonyl acetate	Hong fei	[76]
*CpACX1*	δ-caprolactone and δ-decanolactone	Hong fei	[76]

## Data Availability

Not applicable.
